# Helically Magnetized Plasma: From Photonic Fermi‐Arc Metal to Chirality‐Free Uniaxial Medium

**DOI:** 10.1002/nap2.70035

**Published:** 2026-02-25

**Authors:** Wanxia Huang, Jinyu Hou, Maosheng Wang, Lei Zhou, Shaojie Ma

**Affiliations:** ^1^ College of Physics and Electronic Information Anhui Normal University Wuhu China; ^2^ State Key Laboratory of Surface Physics, Key Laboratory of Micro and Nano Photonic Structures (Ministry of Education) and Department of Physics Fudan University Shanghai China; ^3^ Shanghai Key Laboratory of Metasurfaces for Light Manipulation Shanghai China; ^4^ Department of Optical Science and Engineering, College of Future Information Technology Fudan University Shanghai China

**Keywords:** fermi‐arc metal, magnetized plasma, Weyl semimetal

## Abstract

Fermi‐arc metals, unconventional semi‐metals featuring cylindrical Fermi surfaces formed by Fermi arcs, have recently attracted extensive attention for realizing a novel metallic phase that retains chiral anomaly responses yet suppresses quantum oscillations. Although it was proposed that spatially twisting a superlattice of thin Weyl metals can form a Fermi‐arc metal, previous local‐approximation analyses are valid only for slowly varying systems and cannot capture all rich physics in such systems. Here, we report an optical realization of such a phase in a natural magnetized plasma subjected to a helically modulated magnetic field. Unlike previous studies on artificial heterostructure platforms, we admit a fully analytical, nonperturbative treatment that tracks a complete evolution of Weyl node. In the slowly varying regime, our platform faithfully realizes and manipulates the Fermi‐arc metal state in a real system. As the modulation rate increases, Fermi‐arcs inheriting opposite chiralities start to hybridize. Remarkably, in the deep nonperturbative limit, chirality vanishes, not through the conventional Weyl point annihilations, but via Fermi arc recombination, resulting in a chirality‐free uniaxial optical medium. These findings unveil global topological transitions in nonuniform Weyl systems and open routes toward photonic devices based on engineered Fermi‐arc dynamics.

## Graphical abstract

A helically modulated magnetic field in magnetized plasma realizes a photonic Fermi‐arc metal, enabling full analytical tracking of Weyl node evolution. As modulation strengthens, opposing‐chirality Fermi arcs hybridize and recombine, yielding a chirality‐free uniaxial optical medium without conventional node annihilation. This reveals global topological transitions in nonuniform Weyl systems and unveils new pathways for photonic devices harnessing engineered Fermi‐arc dynamics.

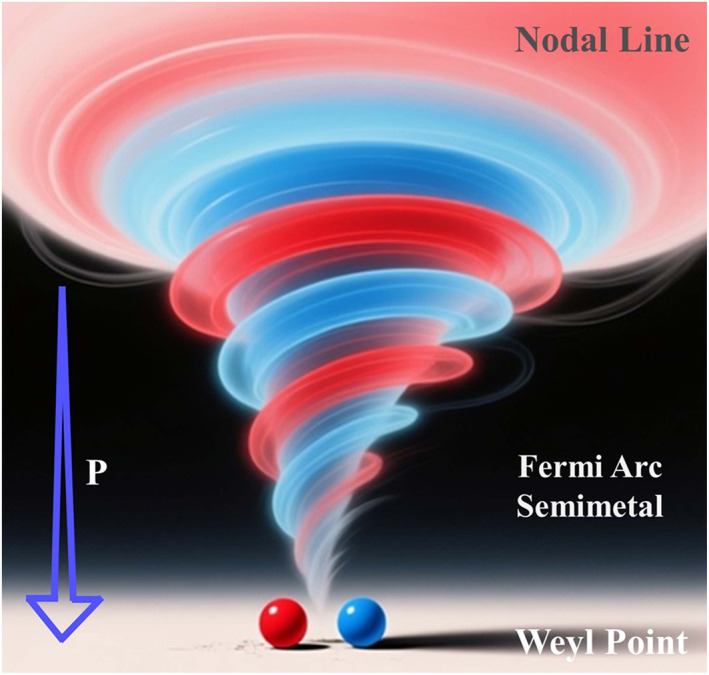

## 1 Introduction

Weyl semimetals [1, 2, 3] exhibit topologically nontrivial degeneracies characterized by twofold linear band crossings, known as Weyl points (WPs) [4, 5]. These degenerate points behave similar to massless Weyl fermions, carrying quantized Chern numbers $c_{1}=\pm 1$ [6, 7] and remaining robust under generic perturbations. In crystalline systems, WPs always emerge in pairs with opposite chirality, and annihilation only occurs when oppositely charged WPs converge and collide. These bulk topological features give rise to extraordinary surface phenomena known as Fermi arcs [8, 9], where topological protected surface mode connect the projection of opposite WPs. Owing to the nontrivial bulk topological Chern numbers, Weyl semimetals exhibit a variety of unconventional electromagnetic (EM) responses, such as chiral anomaly [10, 11], quantum oscillations [12, 13], negative magnetoresistance [14, 15, 16], negative refraction [17], and bulk‐edge correspondence [18], as being subjected to external electric and magnetic fields.

Recently, inhomogeneous systems composed of semi‐metals with spatially varying WP properties have attracted significant attention, as unusual electromagnetic responses can arise from their constituent sub‐systems [19, 20]. In particular, recent theoretical studies based on tight‐binding models have revealed that a superlattice formed by spatially twisting thin Weyl‐metal layers host a distinctive semimetallic phase, termed the Fermi‐arc metal [20, 21, 22, 23, 24], in which the low‐energy spectrum is entirely composed of Fermi arcs, and chiralities of WPs are inherited by a series of spatially separated Fermi‐arc modes. These Fermi arcs collectively form a cylindrical Fermi surface that preserves the chiral anomaly, giving rise to a unique metallic state that already reaches an ultra‐quantum regime at zero magnetic field, characterized by universal low‐field ballistic magnetoconductance and the absence of quantum oscillations. Despite of these exciting predictions, however, previous theoretical framework relies on a local Hamiltonian description, which necessitates chiral Fermi‐arc modes to be fully localized and completely mutually decoupled. Unfortunately, in extreme regimes, inter‐arc coupling may emerge, giving rise to phenomena beyond the scope of such a local model.

In a parallel line, significant effects were devoted to designing artificial photonic systems exhibiting extraordinary topological properties originally predicted in electron systems. For example, Weyl‐type band structures have been realized in carefully designed photonic crystals [25, 26], metamaterials [27, 28], magnetic plasma [29], waveguide arrays [30, 31], and ring‐resonator lattices [27, 32]. These studies have provided a versatile platform for guiding the design of photonic devices to manipulate EM waves. However, the novel Fermi‐arc metallic phase has not yet been realized in photonic systems, let alone the rich physics that may emerge in such systems.

In this article, we propose an optical realization of Fermi‐arc metals using a naturally magnetized plasma subjected to a helically modulated magnetic field. Free from near‐field effects in heterostructures, this system enables a clean first‐principles description via plane‐wave expansion and captures the full evolution from slow to rapid twist modulation, thus providing an ideal platform to explore the related topological physics. In the slowly varying regime, we find that this platform provides a robust framework for precise realization and tunable control of the Fermi‐arc metal state in real systems, in consistency with predictions by the local model. As the spatial modulation strengthens, Fermi arcs originating from WPs with opposite chiralities begin to interact and hybridize, which drives nontrivial recombination processes that invalidate the local‐model description. Remarkably, in the deep nonperturbative regime, the system undergoes a topological transition in which the net chirality vanishes, not through the conventional WPs annihilation as predicted by previous theories, but via a recombination of spatially separated Fermi‐arc modes. This process leads to the emergence of a uniaxial optical medium that is effectively chirality‐free, despite of originating from a magnetic Weyl system. These results shed lights on the global topological evolution of nonuniform Weyl superstructure and open new avenues for designing photonic platforms where Fermi arc dynamics, rather than bulk band topology alone, dictate the optical responses.

## 2 Photonic Band Structure in a Helically Magnetized Plasma

**FIGURE 1:**
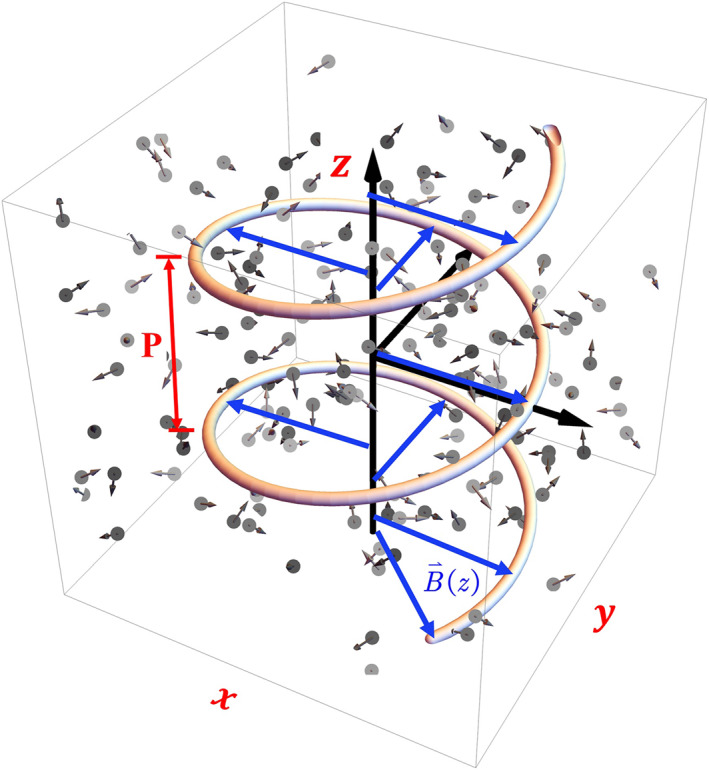
Schematic of a helically magnetized plasma, formed by a natural plasma subjected to a helically modulated magnetic field $\vec{B}\left(z\right)$.

The helically magnetized plasma, obtained by placing a natural plasma in a helically modulated magnetic field $\vec{B}\left(z\right)$, is shown in Figure 1. Brown helical curves depict the direction of the spatially varying horizontal external magnetic field:

(1) $$\vec{B}\left(z\right)=B_{C}\cdot \left[\cos\left(\Lambda \cdot z\right)\cdot \hat{x}+\sin\left(\Lambda \cdot z\right)\cdot \hat{y}\right],$$

where we have Λ=2π/P, with P being the pitch length. Such a magnetic‐field configuration can be realized using carefully designed helically twisted coils (details of the implementation are provided in Supporting Information S1) [33]. The modulation is characterized by two normalized frequencies: the plasma frequency $\omega_{p}$ and the cyclotron frequency $\omega_{c}\propto B_{C}$, with proportionality set by the charge‐to‐mass ratio q/m. For simplicity, all fundamental constants, such as the elementary charge q, particle mass m, and speed of light c, are set to unity in the discussions followed.

We compute the photonic band structure of this periodically twisted system by tracking the propagation of an EM plane wave inside the system. Electric field inside the EM wave excites the magnetized plasma, and the induced polarization feeds back into Maxwell's equations, leading to a set of self‐consistent coupled equations to describe the EM wave propagations. We exploit the periodicity along the z‐direction to expand all EM fields by a set of plane‐wave bases, yielding $9\cdot \left(2N_{\max}+1\right)$ coupled eigen equations with $N_{\max}$ being the maximum plane‐wave order. Solving these equations numerically yields the photonic band structure of our system. This formulation corresponds to the standard plane‐wave expansion method (PWEM). It provides a clean and first‐principles description of the helically magnetized plasma across the full range of twist modulation velocities, yielding an ideal platform to explore its spectral evolution and transition properties (see Supporting Information S1 for details).

**FIGURE 2:**
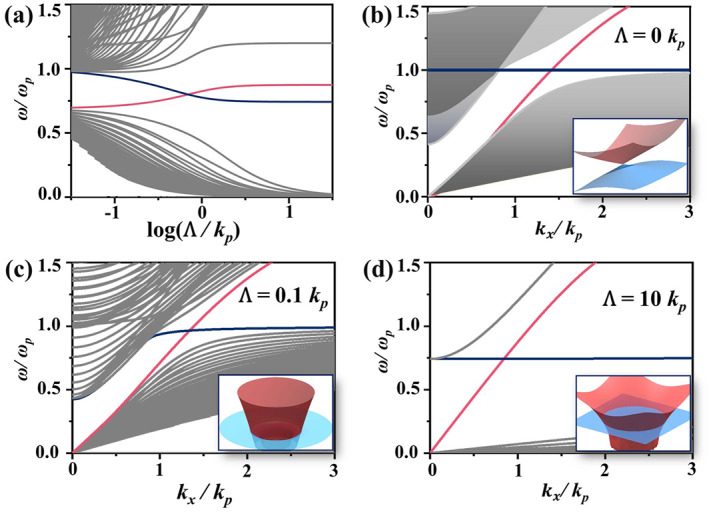
Evolution of the photonic band structure under the influence of twist. (a) Band structure evolution as a function of twist velocity Λ, with fixed parameters $\omega_{c}=2\omega_{p}$, $k_{x}=k_{p}$, and $k_{y}=k_{z}=0$. The isolated modes near the original WP are highlight in red and blue, corresponding to the LCP and LM mode, respectively. (b–d) Dispersion curves along $k_{x}$ for selected twist velocities: (b) Λ=0, (c) $\Lambda =0.1k_{p}$, and (d) $\Lambda =10k_{p}$, respectively. The insets in panels (b–d) show the corresponding dispersion surfaces formed by the two isolated CZM bands in $k_{x}-k_{y}-\omega$ space.

Figure 2a displays the calculated photonic band structure as a function of the twist modulation velocity, denoted by $\log\left(\Lambda /k_{p}\right)$, with $k_{p}=\omega_{p}/c$. In this twist‐periodic system, Bragg scatterings induce strong intermodal coupling, giving rise to a complex photonic band structure evolution. With increasing Λ, the dispersion relations of most modes become intertwined and shift in opposite frequency directions, whereas a few isolated modes, highlighted in red and blue, stand out near the center of the spectrum. Although these isolated modes may appear to cross at certain points, such intersections are accidental and do not correspond to any meaningful topological transition. In the extreme limit $\Lambda \gg k_{p}$, these isolated modes become entirely independent of Λ, indicating that they are effectively decoupled from the multiple scattering processes that govern the rest of the spectrum. To comprehensively illustrate the evolution of the band characteristics, we plot the dispersion relations for several selected twist modulation velocities, Λ=0, $0.1k_{p}$, and $10k_{p}$, as shown in Figure 2b–d. These results reveal the evolution from the conventional Weyl cone behavior through the cylindrical Fermi‐arc metal phase to a chirality‐free uniaxial optical medium, highlighting the critical role of modulation strength in shaping the system's topological properties.

We first consider the limiting case of Λ=0, where the system reduces to the well‐known magnetized Weyl semimetal modeled as a plasma under a uniform magnetic field $\vec{B}=B_{C}{\hat{e}}_{x}$. For a strong magnetic field satisfying $\omega_{c}>\omega_{p}$, pairs of WPs emerge due to the hybridization of plasma resonance and circularly polarized modes. The local dispersion near the outer WPs, located at $\vec{k}=\left[\pm K_{r},0,0\right]$ and $\omega =\omega_{p}$ with $K_{r}=\omega_{p}/c\cdot \sqrt{\omega_{c}/\left(\omega_{c}-\omega_{p}\right)}$, can be captured by an effective local Hamiltonian derived using a k·p perturbative approach [34]:

(2) $$H=\omega_{p}+\chi \cdot \left[v_{\|}\left(k_{x}-\chi K_{r}\right)\left(\sigma_{x}+\sigma_{0}\right)+v_{⊥}k_{y}\sigma_{y}+v_{⊥}k_{z}\sigma_{z}\right],$$

with χ=±1 denoting the chirality of the WPs, and where $v_{\|},v_{⊥}>0$ represent the Fermi velocities parallel and perpendicular to the magnetic field, respectively, determined fully by the ratio $\omega_{c}/\omega_{p}$. Two WPs located at $k_{x}=\pm K_{r}$ possess opposite chiralities. These bulk modes vary smoothly with $k_{y}$ and $k_{z}$, forming a continuous spectrum region depicted in Figure 2b.

We next consider the cases of non‐zero Λ. At slow modulation speed with $\Lambda =0.1k_{p}$, strong intermodal coupling is introduced, resulting in a complex band structure whose mode spacing scales with Λ, as depicted in Figure 2c. Isolated modes persist near the original WP locations at $k_{x}=\pm K_{r}$, where they intersect and retain distinct spectral signatures. In contrast, under a rapid twist with $\Lambda =10k_{p}$, these intricate intersection features gradually vanish, leaving only a few discernible modes within the computational region, as illustrated in Figure 2d. A more detailed analysis of each regime will be presented in the following sections. Notably, due to the underlying helical symmetry, the degenerate points observed along the $k_{x}$‐direction in Figure 2c,d are not isolated but extend across all horizontal directions, forming a line degeneracy rather than discrete points. As illustrated in the inset, which presents a zoomed‐in three‐dimensional local band structure, the two isolated bands exhibit zero‐dimensional degeneracies (WPs) at Λ=0, which continuously evolves into one‐dimensional, line‐like degeneracies for non‐zero Λ. This evolution highlights the fundamental distinction between WPs and extended Fermi‐arc metallic states.

## 3 Photonic Fermi‐Arc Metals in a Slowly Twisted Plasma

In a slowly twisted plasma ($\Lambda \ll k_{p}$), periodic modulation leads to a complex band structure due to intermodal coupling. Despite of these complicities, we can employ a perturbative approach to analytically study the emergence of the Fermi‐arc metal states in this spatially modulated plasma, focusing on the regions near two original WPs. Specially, the slow spatial variation of the magnetic‐field direction shifts WP positions between neighboring regions, acting as an effective field that induces chiral zero modes appearing as Fermi‐arc boundary states connecting the projected WPs.

**FIGURE 3:**
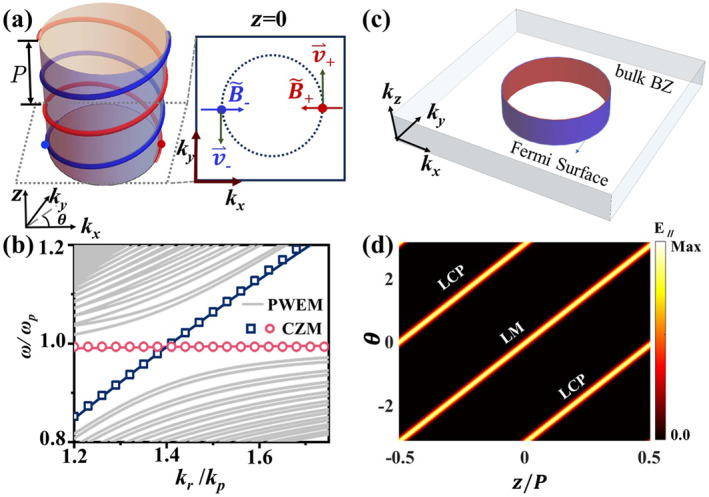
Fermi arc semimetal in a slowly twisted magnetic plasma. (a) Evolution of WPs in the helically magnetized plasma. The inset shows a cross‐section view at *z* = 0, illustrating the local orientation of the emergent gauge field relative to the WP distribution. (b) Band structure from global Hamiltonian (solid lines) and localized Hamiltonian (symbols), with fixed parameters $\omega_{c}=2\omega_{p}$ and $\Lambda =0.02k_{p}$. (c) Bulk Brillouin zone with chiral Fermi surfaces, where red and blue surface possess positive and negative chirality, respectively. (d) Heatmaps of the chiral eigenstates, as a function of the orientations θ in momentum space at fixed $K_{r}=\sqrt{2}\omega_{p}/c$. Here, “LCP” and “LM” stand for the transverse and longitudinal chiral modes, respectively.

We take the Weyl degeneracy in a uniform magnetic field as the perturbative starting point to examine mode evolution under a spatially varying field. Under the lowest‐order quasi‐steady approximation, WPs align their orientation with the direction of the external magnetic field, as given by Equation (2). Due to the helical configuration of the external magnetic field, WPs with opposite chirality form a double‐helix structure in the $k_{x}{\;-\;k}_{y}-z$ space, as represented by the red and blue helical curves in Figure 3a. Positions of these WPs at a given z‐location are described by

(3) $${\vec{K}}_{\text{WP}}\left(z\right)=\chi \cdot \left[K_{r}\;\cos\left(\Lambda \cdot z\right),K_{r}\;\sin\left(\Lambda \cdot z\right),0\right].$$

Two opposite WPs are always symmetrically positioned at $\pm {\vec{K}}_{\text{WP}}$. Their motion along the *z*‐direction can be modeled as an effective position‐dependent gauge field $\vec{A}\left(z\right)$, which locally shifts the position of the WPs. This gauge field introduces a corresponding effective local magnetic field that couples to the helically distributed WPs, thereby modulating their behavior [20]:

(4) $${\vec{\tilde{B}}}_{\chi }=\nabla \times {\vec{K}}_{\text{WP}}=-\chi \Lambda K_{r}\cdot \left[\cos\left(\Lambda \cdot z\right),\sin\left(\Lambda \cdot z\right),0\right]=-\Lambda \cdot {\vec{K}}_{\text{WP}}.$$

This emergent magnetic field is always perpendicular to the WP trajectory $\vec{v}=\partial {\vec{K}}_{\text{WP}}/\partial z$ and aligns parallel to their local displacements, as shown in the inset of Figure 3a on a representative plane at *z* = 0.

This magnetic field gives rise to characteristic Landau quantization, producing discrete energy levels and chiral modes. For an isolated WP, the gauged Weyl system supports nearly symmetric high‐order Landau levels and a distinctive chiral zero mode (CZM) [35, 36, 37, 38], whose group velocity is governed by both the magnetic‐field direction and the chirality of the WP. The CZM inherits the chiral character of the WP, predominantly confined to a single basis component. A similar chiral mode emerges in the plasma with helically modulated WPs. Specially, the local dispersion of the modulated WPs with $k_{r}=K_{r}$ can be analytically expressed as

(5) $$E_{+}=\omega_{p},E_{-}=\omega_{p}+2v_{\|}\left(k_{r}-K_{r}\right),$$

where $k_{r}=\sqrt{k_{x}^{2}+k_{y}^{2}}$ denotes the magnitude of the in‐plane wavevector. The red circles and blue squares in Figure 3b depict the analytically predicted CZM dispersion originating from two WPs with opposite chirality, where red denotes the longitudinal mode (LM) and blue the left‐handed circularly polarized mode (LCP). The low‐energy spectrum is composed solely of extended CZMs, placing the system in an extended ultraquantum‐like regime. These predictions exhibit excellent agreement with the numerical results derived from PWEM. From a discrete perspective, this continuously varying helical structure can be viewed as a stack of Weyl semimetal layers undergoing incremental in‐plane rotation. Therefore, these CZMs can also be interpreted as Fermi arc surface states connecting adjacent layers of Weyl semimetals, effectively linking the projected WPs of identical chirality, consistent with predictions of Fermi‐arc metals from the previous tight‐binding model [20].

These chiral modes, similar to the CZM, are dominated by a single basis state and are strongly localized along the z‐direction. Near a representative WP, these chiral modes exhibit Hermite‐Gaussian spatial profiles, centered around $\pm {\vec{K}}_{\text{WP}}\left(z_{0}\right)$ in momentum space and $z=z_{0}$ in real space:

(6) $$\psi_{0}=c_{0}\cdot \exp\left[-2\pi^{2}\cdot \frac{K_{r}}{\Lambda }\cdot {\left(\tilde{z}-{\tilde{z}}_{0}\right)}^{2}\right],$$

where $c_{0}$ is the normalization factor and ${\tilde{z}}_{0}=z_{0}/P$ represent the relative position along the helix. In the adiabatic limit Λ→0, this field becomes increasingly localized relative to the pitch length, asymptotically approaches a δ(z)‐like profile. Consequently, the corresponding iso‐frequency surface becomes nearly dispersionless along $k_{z}$ within the reduced Brillouin zone, forming two nearly overlapping quasi‐continuous cylindrical shells in momentum space, as shown in Figure 3c, which are topologically equivalent to the continuous cylindrical Fermi surfaces in the electronic theory [20]. Each branch arises from an isolated WP helix structure and exhibits a characteristic chiral field profile inherited from the chirality of the constituent WPs. The angular orientation of this localized field in the horizontal momentum space gradually varies with the position along the *z*‐direction, as shown in Figure 3d. Notably, the observed pattern includes contributions from both WPs with opposite chirality. At the same *z*‐position, their orientations in momentum space differ by an angle of π and the two branches are centered at $\pm K_{\text{WP}}$, respectively. Meanwhile, co‐propagating chiral branches with identical angular orientation occur at *z*‐positions separated by half a helical pitch, P/2. Due to the spatial separation, these chiral modes originating from different WPs are effectively decoupled, validating the accuracy of the local approximation.

**FIGURE 4:**
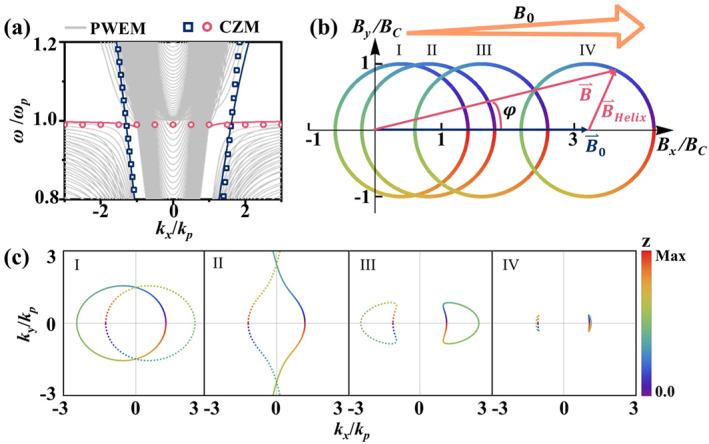
Evolution of the fermi arc semimetal under an additional uniform magnetic field $B_{0}$. (a) Representative dispersion curves of the helically magnetized plasma with an additional uniform magnetic field along the *x*‐direction, with fixed parameters $\Lambda =0.02k_{p}$, $\omega_{C}=2\omega_{p}$, and $B_{0}=0.2B_{C}$. (b) Schematic illustration of the horizontal projection of the total magnetic field $\vec{B}\left(z\right)={\vec{B}}_{\text{Helix}}\left(z\right)+B_{0}\cdot {\hat{e}}_{x}$ for varying values of $B_{0}$. (c) Trajectory of WP positions under increasing $B_{0}=0.4B_{C}$, $0.8B_{C}$, $1.6B_{C}$, and $3.2B_{C}$, corresponding to the selected position indicated by the dashed line in (b).

A similar local approximation and interpretation of the chiral modes can be extended to a broad class of slowly twisted plasmas, even when the magnetic field deviates from a perfectly helical profile. In the following, we examine a scenario where an additional uniform magnetic field $B_{0}$ is applied along the *x*‐direction. Figure 4a shows a representative dispersion relations, with analytically predicted bands based on the local approximation indicated by markers matching well with numerical solutions, revealing an asymmetric band structure characteristic of the Fermi‐arc semimetal. Under the additional uniform magnetic field, the horizontal projection of the magnetic field vector $\vec{B}\left(z\right)={\vec{B}}_{\text{Helix}}\left(z\right)+B_{0}\cdot {\hat{e}}_{x}$ traces an off‐centered circle, with its magnitude varying as a function of *z*, as shown in Figure 4b. This varying magnetic field thereby shifts the local WP in the $k_{x}{-k}_{y}-z$ space, affecting both its orientation and radial position. Under the lowest‐order quasi‐steady approximation, the trajectories of WPs can be theoretically mapped for given parameters, as shown in Figure 4c by the colored solid (positive chirality) and dashed (negative chirality) lines, with color indicating the z‐position. As $B_{0}$ increases, the originally coincident WPs of opposite chirality, separated by P/2, begin to spatially separate in their projected positions. With further increase of $B_{0}$, the total magnetic field becomes weak at certain angles, causing the corresponding WPs to disappear at infinity. As $B_{0}$ continues to grow, the entire magnetic field projection shifts to one side of the origin, and the chiral branches associated with different WPs no longer overlap but instead appear on opposite sides of momentum space. With increasing $B_{0}$, the influence of the helical modulation gradually diminishes, leading to a reduction in the area enclosed by the WP branches. Eventually, in the limit of infinite large $B_{0}$, the system approaches the uniform‐field case, where the enclosed area of the WP branches vanishes, and the states behave as point‐like WP degeneracies.

The WP trajectories evolving along *z*, following a logic similar to that discussed above, give rise to a Fermi‐arc metal state. Due to the z‐dependent varying positions, these WPs are subject to effective magnetic fields ${\vec{\tilde{B}}}_{\chi }=\nabla \times {\vec{K}}_{\text{WP}}$ that induce localized CZM. These chiral modes manifest as Fermi‐arc metal states in momentum space, with projections just coinciding with the original WP locations, similar to the case of a perfectly helical magnetic field distribution. Each branch exhibits a distinct chirality, determined by both the chirality of the corresponding WP and the direction of the effective magnetic field induced by the helical rotation. By slowly tailoring the spatial variation of the magnetic field, magnetized plasmas can support nearly arbitrary distributions of Fermi‐arc metal states, enabling precise control of their modal behavior and governing optical responses through Fermi‐arc dynamics rather than bulk topology alone.

## 4 Photonic Uniaxial Medium in a Rapidly Twisted Plasma

Although the local approximations hold in the slowly twisted regime, where twist‐induced CZMs dominate the Fermi‐arc semimetal response, a broader understanding requires examining the system's evolution as Λ increases, beyond the scope of the previous TBM model. Nevertheless, this regime can still be accurately captured within our framework. As the twist velocity increase, strong couplings emerge between different branches, and eventually in the large Λ limit, the recombination of Fermi arc modes leads to a uniaxial chirality‐free homogeneous medium.

**FIGURE 5:**
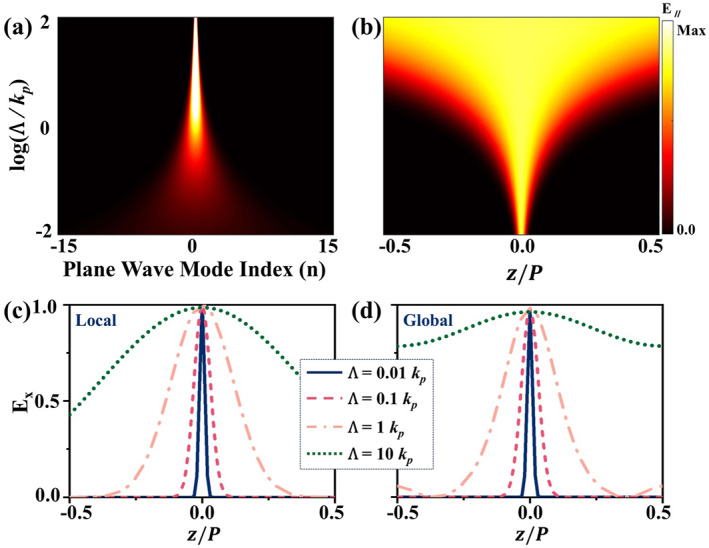
Evolution of the localized CZM with varying Λ. (a, b) Distribution of CZM eigenstates shown (a) in the plane‐wave mode basis and (b) in real space, calculated using the localized approximations, plotted as functions of $\log\left(\Lambda /k_{p}\right)$. (c, d) Four representative real‐space field profiles, computed via (c) the localized approximations, and (d) the full global PWEM.

The system's behavior at various Λ values is first verified numerically, with Figure 5a,b depicting the evolution of the chiral modes. In the slowly twisted limit, where the local approximations remain valid, strong coupling between plane‐wave components gives rise to broad excitation across momentum space and the formation of a spatially localized Fermi‐arc mode. As Λ increases, the spatial localization within each period gradually weakens, as predicted by Equation (6), accompanied by reduced coupling to plane‐wave components. With further increase of Λ, only a few plane‐wave modes are excited, and the field becomes fully delocalized over the modulation period. In this regime, chiral branches originating from different WPs begin to overlap and couple to each other due to the extended nature of their wavefunctions, rendering the local approximation inadequate to describe the system's response. This discrepancy is highlighted in Figure 5c,d, which numerically compare mode profiles computed via the local approximation and the full global plane‐wave expansion for various Λ values. As Λ increase, the Fermi‐arc modes in the local modal broaden, and inter‐branch coupling grows, causing the local approximation to progressively deviate from the exact mode distribution and leading to a pronounced amplitude enhancement near the midpoint |z/P|≈0.5.

This coupling evolution can be captured analytically using higher order perturbative methods, with the local response near $k_{r}=K_{r}$ described by

(7) $$H\left(k_{r},k_{z},\Lambda \right)=\left[\begin{array}{ll} \omega_{p}+2v_{\|}\left(k_{r}-K_{r}\right)+t_{11}+2{\tilde{t}}_{11}\cdot \cos\left(k_{z}P\right) & t_{12}\cdot \left(1+e^{-ik_{z}P}\right)\\ {t_{12}}^{*}\cdot \left(1+e^{+ik_{z}P}\right) & \omega_{p} \end{array}\right],$$

where $t_{11}\propto \Lambda$, ${\tilde{t}}_{11}\propto \left(\Lambda +2\pi^{2}K_{r}\right)\cdot \exp\left(-\pi^{2}K_{r}/\Lambda \right)$, and $t_{12}\propto \left(6\Lambda +\pi^{2}K_{r}\right)\times$ $\exp\left(-\pi^{2}K_{r}/4\Lambda \right)$ represent the coupling terms between CZMs located at distinct positions. At small Λ, all coupling terms are negligible, yielding two independent sets of CZM modes with $k_{z}$‐independent dispersion. As Λ increase, intermodal coupling arises, linking Fermi‐arc modes of opposite chirality and introducing $k_{z}$‐dependent dispersion. These Fermi arcs progressively overlap and hybridize, continuously suppressing local topological signatures such as Berry phase induced chirality. In the limit Λ→∞, this inter‐branch coupling ultimately drives the recombination of Fermi‐arc modes with opposite chiralities, eliminating the system's effective chirality and triggering a topological phase transition. In this regime, the energy separation between different Bloch orders in PWEM diverges, leaving only a few low‐order modes to contribute to the low‐energy response. Simultaneously, the helical modulation becomes deeply subwavelength compared with the wavelength of the low‐energy modes, where the plasma effectively behaves as a homogenized medium. Consequently, Bragg scattering is suppressed, and the system becomes insensitive to the twist, as illustrated in Figures 2a and 5a in the large Λ regime.

To describe the response in this regime, we start from the empty‐lattice approximation, retaining only Bragg scattering, and identify all zero‐energy modes to serve as a complete basis for approximating the full system. This procedure leads to a global, Λ‐independent Hamiltonian, in which higher‐order diffraction processes are recursively folded into the zeroth‐order term, yielding an effective medium description of the system [39]. In the rapidly twisted helical external magnetic field, the plasma behaves as a uniaxial medium with a diagonal permittivity tensor:

(8) $$\varepsilon =\left[\begin{array}{ccc} \varepsilon_{t} & 0 & 0\\ 0 & \varepsilon_{t} & 0\\ 0 & 0 & \varepsilon_{z} \end{array}\right],$$

where $\varepsilon_{t}=1-\frac{\omega_{p}^{2}}{\omega^{2}}-\frac{{\omega_{c}}^{2}{\omega_{p}}^{2}}{2\omega^{2}\left(\omega^{2}-{\omega_{c}}^{2}-{\omega_{p}}^{2}\right)}$ and $\varepsilon_{z}=1-\frac{\omega_{p}^{2}}{\omega^{2}-\omega_{c}^{2}}$. Rapid twisting quenches the off‐diagonal terms from the local magnetic field, thereby eliminating gyrotropic behavior. Remarkably, the chiral features in magnetized plasmas, associated with WPs, are canceled out through strong coupling between modes of opposite chiralities. This induces a topological transition from a chiral to an achiral photonic phase, which arises not from the conventional WP annihilation, but from the recombination of Fermi‐arc modes. As a result, this evolution is not associated with a sharply defined topological transition point in conventional topological systems, but instead proceeds through a smooth crossover process.

**FIGURE 6:**
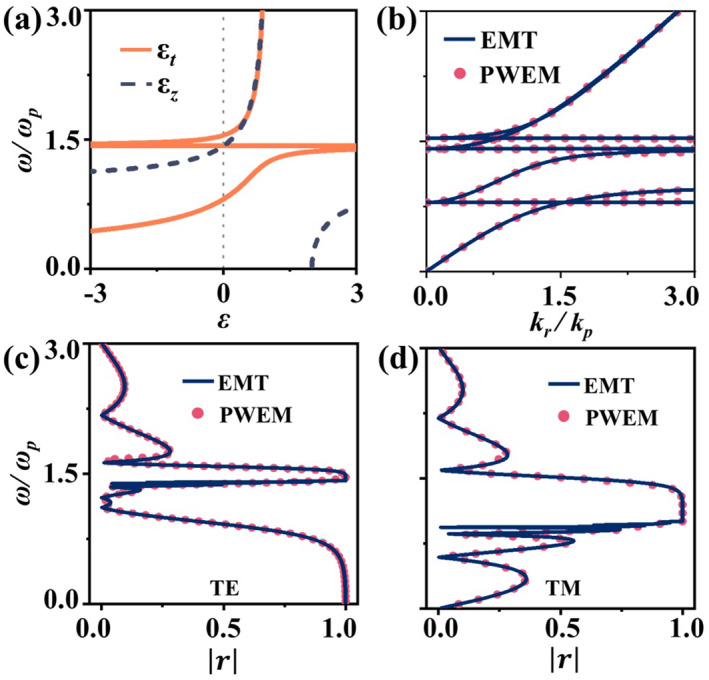
Electromagnetic response of the uniaxial medium under a rapidly twisted helical magnetic field. (a) Frequency‐dependent effective permittivity dispersion in the transverse plane and along the *z*‐direction. (b) Representative band structure and (c, d) reflectance amplitude spectra of the homogeneous uniaxial medium for (c) TE and (d) TM polarizations, comparing results from the EMT model (lines) with global PWEM solutions (circles). The strength of the helical magnetic field is fixed at $\omega_{c}=\omega_{p}$.

This effective medium accurately captures the system's behavior in the Λ→∞ limit. The dispersion of the resulting homogeneous medium is shown in Figure 6a. Besides the intrinsic Drude‐type resonance of the electron gas, the system exhibits additional resonant frequencies at $\omega =\omega_{c}$ along the *z*‐direction and $\omega =\sqrt{{\omega_{c}}^{2}+{\omega_{p}}^{2}}$ in the transverse plane, both tunable by the strength of the external magnetic field. For this uniaxial medium, the internal modes have exact analytical solutions. Their dispersion, illustrated in Figure 6b, perfectly matches rigorous PWEM solutions, revealing tunable nodal line behavior when $\omega_{c}>\sqrt{2/3}{\cdot \omega }_{p}$ (see Supporting Information S4 for details), with both the position and frequency of the Nodal line controllable by the magnetic field. This consistency extends to reflectance and transmittance spectra [39, 40], where the effective medium model (EMT) accurately captures the plasma system's external response, as shown in Figure 6c,d. These results collectively demonstrate that in the limit Λ→∞, the EMT offers a comprehensive description of the plasma. In this regime, the system loses the chirality characteristic but behaves purely as a homogeneous uniaxial crystal.

## 5 Conclusion

This study develops a rigorous theoretical framework to investigate an electron plasma subjected to a helically modulated magnetic field, enabling a transition from a photonic Fermi‐arc metal to a chirality‐free uniaxial medium. In the regime of slowly rotating magnetic fields, the Fermi surface is dominated by CZMs originating from magnetized WPs in motion. As the modulation velocity increases, the system undergoes an unconventional topological transition: the net chirality vanishes not through WP annihilation but via recombination of spatially separated Fermi arc states. This transition leads to an effectively chirality‐free uniaxial optical medium. Our findings elucidate the global topological evolution in nonuniform Weyl superstructures and establish a novel platform for exploring photonic systems dominated by Fermi arc dynamics.

## Author Contributions

S.M. conceived the initial concept, W.H. and J.H. performed numerical simulations and analytical calculations, M.W., L.Z., and S.M. participated in the analysis of the results, and L.Z. and S.M. supervised the project.

## Funding

This work was supported by National Key Research and Development Program of China (Grants 2023YFA1406901, 2023YFA1407700, and 2022YFA1404700), National Natural Science Foundation China (Grants 12374343, 12221004, 62192771), Natural Science Foundation of Anhui Province (Grant 2108085MA23), the State Key Laboratory of Surface Physics Fudan University (Grant KF2023_05) and the Start‐Up Funding of Fudan University (Grant JIH1232133Y).

## Conflicts of Interest

The authors declare no conflicts of interest.

## Supporting information


Supporting Information S1

## Data Availability

All data are available in the main text or Supporting Information S1.
