# Supplementary material for: Helically Magnetized Plasma: From Photonic Fermi‐Arc Metal to Chirality‐Free Uniaxial Medium
Source: Nanophotonics. 2026 Feb 25;15(5):e70035. doi: 10.1002/nap2.70035 (PMC12965010; doi:10.1002/nap2.70035)
Supplement: Supplementary file 1 — Supporting Information S1 [file NAP2-15-e70035-s001.docx]

**Supplementary materials:**

**Helically Magnetized Plasma: from Photonic Fermi-Arc Metal to Chirality-Free Uniaxial Medium**

Wanxia Huang^1*^, Jinyu Hou^1*^, Maosheng Wang^1^, Lei Zhou^2,3†^, and Shaojie Ma^3,4†^

^1^College of Physics and Electronic Information, Anhui Normal University, Wuhu 241000, China

^2^State Key Laboratory of Surface Physics, Key Laboratory of Micro and Nano Photonic Structures (Ministry of Education) and Department of Physics, Fudan University, Shanghai 200438, China

^3^Shanghai Key Laboratory of Metasurfaces for Light Manipulation, Shanghai 200433, China

^4^Department of Optical Science and Engineering, College of Future Information technology, Fudan University, Shanghai 200433, China

*These authors contributed equally to this work.

^†^Correspondence: phzhou@fudan.edu.cn (L.Z.) and shaojiema@fudan.edu.cn (S.M.)

Contents

[S1. Feasibility of Experimental Realization 1](#_Toc218675716)

[S2. Hamiltonian Description of Helically Magnetized Plasma 3](#_Toc218675717)

[S2.1 Hamiltonian of the Magnetized Plasma 3](#_Toc218675718)

[S2.2 Plane Wave Expansion Method for the Helical Plasma 4](#_Toc218675719)

[S3. Local Description Based on the $\boldsymbol{k}\cdot\boldsymbol{p}$ Hamiltonian 6](#_Toc218675720)

[S3.1 Local Hamiltonian Under a Uniform Magnetic Field 6](#_Toc218675721)

[S3.2 Local Hamiltonian Under a Slowly Varying Helical Magnetic Field 7](#_Toc218675722)

[S3.3 Local Hamiltonian Under a Slowly Varying Non-Uniform Magnetic Field 10](#_Toc218675723)

[S3.4 Local Hamiltonian for Nonuniform Magnetic Fields with Increasing Variation Rate 11](#_Toc218675724)

[S4 Effective Medium Description Under a Rapidly Varying Helical Magnetic Field 15](#_Toc218675725)

[S4.1 $\boldsymbol{k}\cdot\boldsymbol{p}$ Method for Modeling Helically Magnetized Plasma 15](#_Toc218675726)

[S4.2 $\boldsymbol{k}\cdot\boldsymbol{p}$ Method for Modeling the Effective Medium 17](#_Toc218675727)

# S1. Feasibility of Experimental Realization


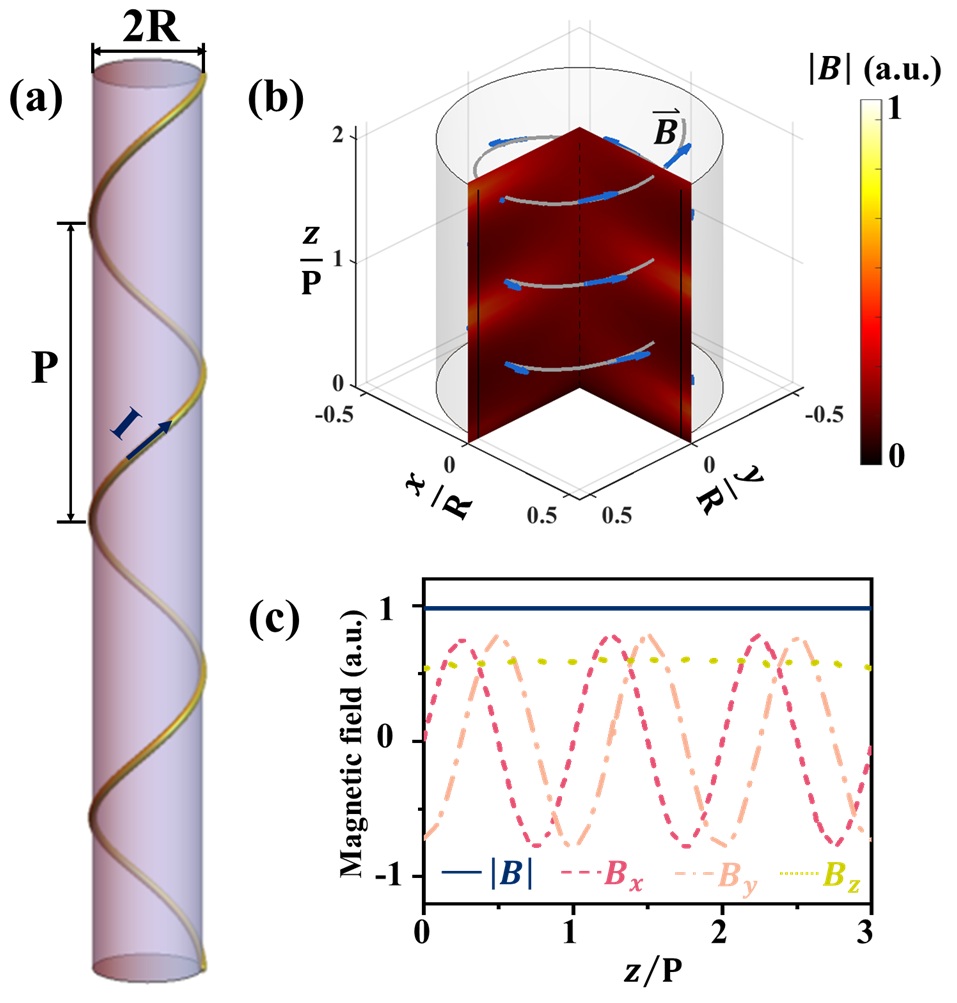


**Figure S1. Schematic diagram of the helical magnetic field.** (a) Schematic illustration of the helically twisted coil configuration used to generate the helical magnetic field. (b) Numerically simulated magnetic-field distributions in three-dimensional space, together with the corresponding cross-sectional $|B|$ profiles on the $xz$ and $yz$ planes. (c) Magnetic-field distributions along the z direction evaluated at $x=y=0$.

In our implementation, a helically varying magnetic field can be generated using helically twisted coils, as illustrated in Fig. S1(a). The spatial variation rate of the magnetic field is determined by the degree of twisting, while the field strength is controlled by the current applied to the coils. The helical magnetic field is numerically verified using Comsol, and the resulting magnetic-field distributions are shown in Figs. S1(b-c). These results clearly demonstrate that the direction of the helical magnetic-field variation is fully consistent with the requirements of our theoretical model. To ensure that the horizontal magnetic-field components dominate ($B_{x}$, $B_{y}$≫$B_{z}$), the coils are designed to be sparse, with the pitch larger than the coil radius. We note that this practical implementation may introduce a small additional magnetic-field component along the $z$ direction. However, such a component neither affect the essential physics nor the main results discussed in this work and can be readily eliminated by introducing a set of Helmholtz coils arranged along the z direction. Furthermore, based on previous studies [S1], a dilute electron gas can be modeled using an InSb semiconductor. This configuration is therefore theoretically feasible and compatible with realistic experimental conditions, establishing a clear and practical route toward experimental realization of the proposed effect.

# S2. Hamiltonian Description of Helically Magnetized Plasma

## S2.1 Hamiltonian of the Magnetized Plasma

Consider the motion of an electron gas under an external magnetic field, where the magnetic field is expressed as:

(S2.1)

In the presence of this external magnetic field, electrons experience both the electric field force and the Lorentz force. Thus, the equation of motion is given by:

(S2.2)

Define the electric dipole moment per unit volume as . Then, the equation of polarization takes the form:

(S2.3)

In this equation, the plasmon frequency $\omega_{p}=\sqrt{Nq^{2}/m\varepsilon_{0}}$ and the cyclotron frequency $\omega_{c}=qB_{C}/m$ characterize the collective electron oscillations and magnetic field effects, respectively. The term $\Gamma=\beta/m$ accounts for the plasma damping. is the unit vector of the magnetic field.

By introducing the auxiliary parameter to linearize the standard Maxwell’s equation, we obtain the following equations to describe the magnetized plasma:

(S2.4)

By assuming a steady state with all fields varying as , the Maxwell’s equations can be rewritten as an intrinsic matrix equation:

(S2.5)

In these block matrices, $I_{3\times3}$ denotes the 3-by-3 identity matrix and we also define:

,  (S2.6)

The $z$-dependent magnetic field preserves derivatives, whereas translational symmetry in the x-y plane permits Fourier representation with in-plane wavevectors $k_{x}$ and $k_{y}.$

## S2.2 Plane Wave Expansion Method for the Helical Plasma

To model the electron dynamics in this periodically twisted plasma, we implement a plane wave expansion method adapted to the system's helical symmetry. The wavefunction is decomposed as:

(S2.7)

Here, the coefficient  quantifies the field component amplitude projected onto the $n$-th order plane wave mode, and $k_{z}$ represents the Bloch wave-vector along the helical axis.

Substituting this wavefunction into the Hamiltonian Eq. (S2.5) yields a set of self-consistent coupled equations that provides a first-principles description of the helically magnetized plasma across all twist modulation regime. These equations can be rewritten as a matrix equation:

(S2.8)

In these block matrices, we have defined the following block matrices:

(S2.9)

By appropriately selecting plane-wave cutoff parameters $N_{max}$, Eq. (S2.8) enables accurate calculation of the band structure, eigenmode distributions, and related properties of the helically magnetized plasma across diverse parameter regimes.

**Discussion:**

1. In a helically magnetized plasma with an external magnetic field applied along the *x*-direction, a minor adjustment to $H_{n,n}^{0}$ suffices, leaving the rest of the framework unchanged.

(S2.10)

1. In a helically magnetized plasma with an external magnetic field applied along the z-direction, a similar minor adjustment to $H_{n,n}^{0}$ suffices, leaving the rest of the framework unchanged.

(S2.11)

1. For an arbitrary magnetic field distribution, the system couples exclusively to the Fourier components of $\vec{B}(z)$. Each term $B_{m}$ couples plane-wave orders $n$ and $n\pm m$, differing by $m$.

# S3. Local Description Based on the $\boldsymbol{k\cdot p}$ Hamiltonian

## S3.1 Local Hamiltonian Under a Uniform Magnetic Field

We first consider a plasma subjected to a uniform magnetic field oriented along the *x*-direction. Due to the translational symmetry along the *z*-direction, the system no longer requires a full plane-wave expansion and can be analyzed at a fixed $k_{z}$, which is equivalent to retaining only the zeroth-order mode in the expansion. Under this simplification, the dispersion relation can be rigorously obtained through eigenvalue analysis.

At $k_{y}=k_{z}=0$, the energy level of the transverse mode satisfies:

(S3.1)

Besides, the plasma supports a $k_{x}$-independent longitudinal mode occurring at $\varepsilon_{x}=0$, occurring at the frequency $\omega=\omega_{p}$. By the orthogonality of the polarizations, these two types of modes intersect at:

(S3.2)

These degeneracy points are Weyl points (WPs) and behaves as source and drain of Berry curvature flux lines [S2]. Without loss of generality, we set and focus on the degeneracy point located at $k_{x}=\pm K_{r,-}$.

To better understand these linear degeneracies, we apply $k\cdot p$ theory to obtain the approximate Hamiltonian near the degeneracies. The degeneracy eigenstates and at the degeneracy point take the form:

(S3.3-a)

and

(S3.3-b)

Here, . Thus, expanding to first order in the vicinity of the outer degeneracy point located at $k_{x}=K_{r,-}$, the effective Hamiltonian can be:

(S3.4)

Where $\chi$ is the chirality of the WPs. The three Fermi velocity components at the positive Weyl point are defined as:

(S3.5)

Near the degeneracy point, the Hamiltonian takes the standard form of a WP, except for an additional tilt term proportional to the identity matrix in the first term, which captures the zero-slope longitudinal mode in the dispersion.

## S3.2 Local Hamiltonian Under a Slowly Varying Helical Magnetic Field

The slowly varying non-uniform external magnetic field induces spatial shifts of the WPs, which can be effectively interpreted as a coupling between the WPs and an emergent gauge field, ultimately modifying the modal response near the WPs [S3].

To illustrate this, we consider a plasma subjected to a helical magnetic field. Under the slowly varying approximation, the position of the WPs gradually evolves with the local orientation of the magnetic field along the *z*-direction, and their location at a typical *z*-position can be expressed as：

(S3.6)

The pair of WPs with opposite chirality are always symmetrically positioned at and , corresponding to positive and negative chirality, respectively.

With the breaking of the translational symmetry along the *z*-direction, $k_{z}$ is no longer a good quantum number, and $k_{z}$ is replaced by $-i\partial_{z}$. Consequently, the local Hamiltonian near a representative *z*-position takes the form:

(S3.7)

where . Owing to the helical symmetry, the system’s properties remain nearly invariant along $z$. For a fixed wavevector near $k_{x}\approx K_{r}$ and $k_{y}\approx0$, we perform a detailed analysis in the vicinity of $z_{0}=0$  for the WP with chirality $\chi=+1$:

(S3.8)

and in the vicinity of $z_{0} =P/2$  for the WP with $\chi=-1$:

(S3.9)

Drawing an analogy to the quantum harmonic oscillator model, we introduce the corresponding creation and annihilation operators

(S3.10)

These operators satisfy . Therefore, such Hamiltonians operate on the Hilbert space spanned by the orthonormal Fock states $|n\rangle$, with $n=0,1,2,\ldots$ representing the number of bosonic particles in two orthonormal subspaces. To rigorously solve this Hamiltonian, one can map this quantized Hamiltonian onto a quasi-2D non-Abelian tight-binding model, with a well-defined $k_{x}$ along x-direction and a semi-infinite virtual 1D lattice denoted by ‘$n$’. By solving this model under a certain truncation, the corresponding mode distributions and dispersion relations can be obtained.

For the specific Hamiltonian forms in Eqs. (S3.8)-(S3.9), the solutions are linear combinations of transverse and longitudinal modes whose orders differ by one. Near $z=0$, the horizontal mode has one order lower than the vertical mode, whereas at $z=P/2$, the relation is reversed. Therefore, the general eigenstates take the form:

(S3.11)

Substituting these expressions into the Hamiltonian leads to quantized energy levels. Specially, the ground states are given by:

(S3.12)

Both the dispersion and eigenstate follow only a single branch of the original twofold degenerate Weyl modes, which is referred to as the chiral zero mode (CZM). The WPs located at the same wavevector position but separated by half a period along the z direction acquire opposite chiralities due to the reversed orientation of the magnetic field. Nevertheless, since the rotation direction remains unchanged, the effective gauge field coupled to the WP is preserved, leading to a reversal in the mode selected by the CZM.

The eigenstates of the CZM along the z-direction can be analytically expressed as a zeroth-order Hermite-Gaussian mode:

(S3.13)

These chiral modes exhibit Hermite-Gaussian profiles, centered around in momentum space and $z=z_{0}$ in real space. Here, $\tilde{z}_{0}=z_{0}/P$ represent the relative position along the helix.

Applying a Fourier transform on the Hermite-Gaussian eigenstate yields its momentum-space distribution. A smaller $\Lambda$ corresponds to a mode more localized relative to the modulation period, resulting in a broader momentum-space profile. As $\Lambda$ increases, the modes become progressively delocalized, and their momentum-space distribution narrows accordingly. In this regime, different modes begin to couple with one another, and the local approximation breaks down.

## S3.3 Local Hamiltonian Under a Slowly Varying Non-Uniform Magnetic Field

In the adiabatic regime, the existence of localized CZM does not require an ideal helical magnetic field with uniform strength. This description remains valid under arbitrarily inhomogeneous magnetic field profiles. As a specific example, we consider a plasma subject to a helical magnetic field superimposed with an additional uniform magnetic field along the transverse direction. This superposition causes both the magnitude and orientation of the total magnetic field to vary with $z$. Despite these variations, the adiabatic approximation discussed above still holds.

To elucidate the underlying physical mechanisms, we construct a geometric representation of the total magnetic field composed of a uniform *x*-directional magnetic field and a helical magnetic field , as shown in Fig. 4(b) in the main text. The complex form of the total magnetic field is expressed as:

(S3.14)

For simplicity, we define Λ$\cdot z=\theta$. Thus, the magnitude of the magnetic field in a representative direction is given by:

(S3.15)

Based on the discussion for a uniform magnetic field, the position of the WP at a given azimuthal angle $\varphi$ is determined by the projection of the total magnetic field:

(S3.16)

where . Based on Eq. (S3.16), we can theoretically compute the trajectories of WPs under specified parameters, as illustrated by the colored solid and dashed lines in Fig. 4(c) in the main text. Here, solid lines represent positive WPs, and dashed lines denote negative WPs, and the color of the curves corresponds to the magnitude of *z*.

Owing to their z-dependent positions, these WPs experience effective magnetic fields that give rise to localized CZM. In momentum space, these chiral modes manifest as Fermi arc metallic states, whose projections align with the original WP locations. Each branch carries a distinct chirality, governed jointly by the intrinsic chirality of its associated WP and the direction of the effective magnetic field induced by the helical modulation.

## S3.4 Local Hamiltonian for Nonuniform Magnetic Fields with Increasing Variation Rate

As the variation rate $\Lambda$ increases, the locality of the CZM gradually diminishes, inducing additional coupling between modes localized at different positions. This coupling progressively invalidates the CZMs of the local Hamiltonian, as shown in Fig. 5. Here, using CZMs as basis vectors, we analytically describe the coupling evolution with increasing $\Lambda$.

Coupling arises between CZMs localized at the same wave vector. In a plasma subjected to a perfectly helical magnetic field, at a representative position $k_{x}=K_{r}$ and $k_{y}=0$, the first-order approximation reveals that helical magnetic fields generate a series of CZMs with opposite chirality at $z=P/2+nP$ and $z=nP$ (with $n$ an integer), denoted as $\phi_{A,n}$ and $\phi_{B,n}$, respectively. For the eigenequation Eq. (S2.5), these eigenmodes correspond to the first-order treatment of $\Delta$ matrix at different positions. After mode broadening, the modes begin to capture finer spatial variations of the Δ matrix along $z$, reflecting the increased coupling between locally defined states. By using CZMs $\phi_{A,n}$ and $\phi_{B,n}$ as bases and incorporating higher-order approximations, the evolution of these modes can be accurately described by a tight-binding chain model built upon mode coupling.

Near a representative position at $z=0$, CZM corresponds to the eigenmode of the first-order approximation of the Δ matrix in Eq. (S2.6). In what follows we retain three-order contributions in the expansion of Δ:

(S3.17)

Local Hamiltonians of different orders and can thus be defined through the corresponding expansions of the Δ matrix. The two sets of basis states can be analytically solved based on the previous discussion in Section 3.1-3.2:

(S3.18)

The CZMs $\phi_{A,n}$ located at $z=P/2+nP$ are transverse modes, while $\phi_{B,n}$ located at $z=nP$ are longitudinal modes. These CZMs satisfy the following orthogonality relation:

(S3.19)

Owing to the localized nature of the modes, we assume that coupling occurs only between nearest-neighbor $\phi_{A,n}$ and $\phi_{B,n}$ modes, while higher-order couplings are neglected.

Therefore, the eigenmode can be regarded as a superposition of these two localized modes:

(S3.20)

Thus, the eigenvalue equation $H\left( z \right)\phi\left( z \right)=\omega\phi\left( z \right)$ can be locally expanded around each location points $z=nP$ and $z=P/2+nP$, yielding a 2N-by-2N effective Hamiltonian analogous to a $k\cdot p$ formulation, where N denotes the maximum truncation order:

(S3.21)

The result can be rigorously solved:

(S3.22)

Relative to the uncoupled CZM dispersion, the coupling coefficients $t_{ij}$ grow with increasing $\Lambda$ and asymptotically vanish as $\Lambda\to0$, consistent with the first-order local-approximation limit.

For an infinitely long mode chain, the periodicity allows the above Hamiltonian to be reduced to a 2×2 periodic Hamiltonian:

(S3.23)

At small $\Lambda$, all coupling terms $t_{ij}$ are negligible, resulting in two independent sets of CZM modes that exhibit $k_{z}$-independent dispersion. As $\Lambda$ increases, however, intermodal coupling gradually emerges, bridging Fermi-arc modes of opposite chiralities. This interaction lifts their degeneracy and introduces a pronounced $k_{z}$-dependent dispersion, marking the onset of hybridization between the two chiral sectors.


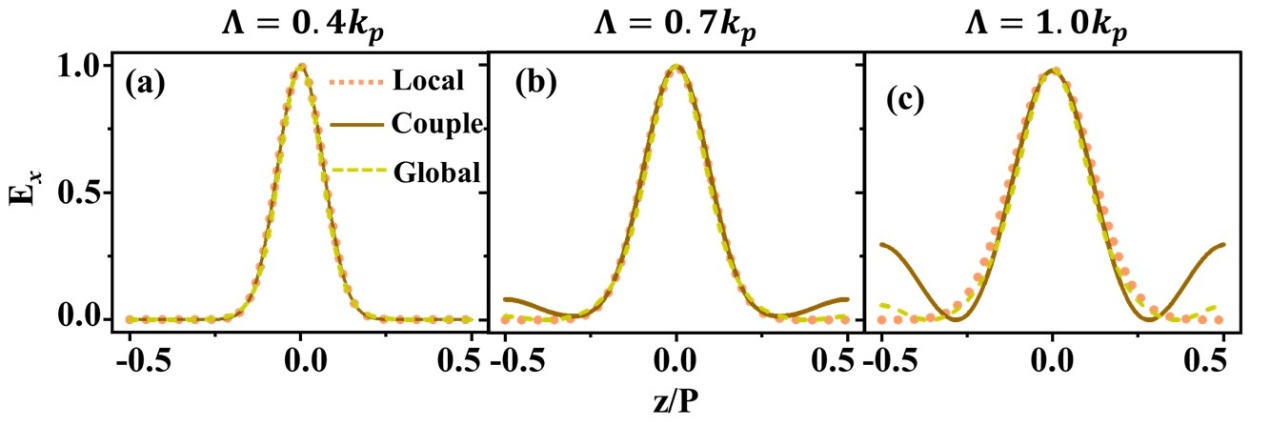


**Figure S2. Spatial lineshapes of the CZM in the Fermi-Arc metal phase.** The tangerine dotted curve represents the first-order local-approximation result neglecting coupling, the dark goldenrod solid curve includes nearest-neighbor coupling, and the dark yellow dashed curve corresponds to the global numerical PWEM calculation.

The numerical results for the coupled model are shown in Fig. S2. In the small-$\Lambda$ limit, the coupling terms $t_{ij}$ are negligible, and the locally approximated CZM modes agree perfectly with those obtained from the rigorous PWEM calculation. As $\Lambda$ increases, a deviation gradually emerges between the two models, yet the coupled model, despite being limited to third-order expansion, still captures the overall broadening behavior, consistent with the theoretical analysis.

As $\Lambda$ increases further, the CZMs delocalize, with more modes across different periods coupling and recombining between opposite chiralities, ultimately forming a chirality-free uniform medium. At this stage, higher-order Δ matrix expansions would be required mathematically, but the local-mode approximation of Eq. (S3.23) breaks down, necessitating a new treatment via the effective-medium model discussed in the next section.

# S4 Effective Medium Description Under a Rapidly Varying Helical Magnetic Field

In the limit $\Lambda\to\infty$, the helical modulation becomes deeply subwavelength compared to the wavelength of the low-energy modes, and the plasma can be effectively treated as a homogenized medium. This effective medium description can be derived using an approach analogous to the $k\cdot p$ approximation, yielding accurate predictions for both the band structure and the transmission/reflection behavior of the system

## S4.1 $\boldsymbol{k}\boldsymbol{\cdot}\boldsymbol{p}$ Method for Modeling Helically Magnetized Plasma

As $\Lambda$ increases, parts of modes described by Eq. (S2.8) diverge and no longer contribute to the low-energy physics. In this regime, we adopt an approach analogous to the empty lattice approximation in electronic systems to identify the set of zero modes that substantially contribute to the low-energy behavior. By using these selected modes as a new basis, we can re-expand the entire system and derive a $\Lambda$-independent effective Hamiltonian, which serves as the foundation for the effective medium description.

Firstly, we divide the Hamiltonian in Eq. (S2.8) into two parts:

(S4.1)

where $H_{0}$ includes all terms that scale linearly with $\Lambda$. The remaining part, denoted as $H_{1}$, contains all other parameters related to the plasma and magnetic field, excluding the rotational velocity $\Lambda$.

The selected modes are the zero modes of the empty Hamiltonian $H_{0}$:

(S4.2)

For a system with plane-wave expansion order ranging from $-N_{\max}$ to $N_{\max}$ , the operator $H_{0}$ yields a total of ${10N}_{\max}+9$ zero modes. Among them, the zeroth-order Bragg mode contributes nine degenerate zero modes to construct a 9-by-9 identify matrix,

(S4.3)

while each non-zero-order mode contributes five degenerate zero modes:

(S4.4)

All of these modes together form a new set of basis vectors $V_{0}$ with a total dimension of .

Using these degenerate basis vectors, the Hamiltonian in Eq. (S2.8) can be projected on to the zero modes $V_{0}$:

(S4.5)

where the superscript “” denotes the conjugate transpose. This yields an effective description that captures only the low-energy modes in the limit $\Lambda\to\infty$. The formula of can be written as a matrix:

(S4.6)

The diagonal block matrices in equation (S4.6) are defined as:

(S4.7)

Meanwhile, the off-diagonal block matrices to describe the coupling are defined as:

(S4.8)

By substituting a specific wavevector $\vec{k}$, the band structure can be obtained through direct numerical diagonalization of .

For a truncation order of $N_{max}=3$, the dispersion can be analytically derived exactly, as given below:

(S4.9)

Here, , which indices that the dispersion is fully rotationally symmetric in the horizontal plane. These band structures are in perfect agreement with that obtained from the full Hamiltonian $H$ using the rigorous plane-wave expansion method, which confirms the validity of the effective model derived from the $k\cdot p$ approach method, as shown in Fig. 6(b) of the main text.

It is noted that the $E_{B-}$ and $E_{C-}$ modes can intersect under certain parameter regimes. Analyzing the $k_{x}\to\infty$ limit reveals that the existence of such a crossing requires that $\omega_{c}\geq\sqrt{2/3}\cdot\omega_{p}$, under which the mode dispersions give rise to a nodal-line structure. This result is consistent with the pair of WPs that emerge in the original system with a uniform magnetic field when $\omega_{c}\geq\omega_{p}$.

## S4.2 $\boldsymbol{k}\boldsymbol{\cdot}\boldsymbol{p}$ Method for Modeling the Effective Medium

Since the system described by excludes Bragg scattering effects and is independent of $\Lambda$, it can be accurately modeled as an effective medium. Mathematically, this corresponds to eliminating all hidden variables associated with higher-order scattering modes, as well as the components of the zero modes projected onto the three $V_{0i}$ basis vectors, via a process of variable elimination. These corresponding basis vectors can be interpreted as auxiliary variables, analogous to the polarization $\vec{\boldsymbol{P}}$ and magnetization $\vec{\boldsymbol{M}}$ in conventional electromagnetic medium.

Formally, after eliminating all terms associated with higher-order Bragg scattering, Eq. (4.6) can be written in the following form:

(S4.10)

Here, the matrix $\bar{A}$ can be rigorously and systematically derived from the matrices $A_{0}$, $A$, $M$, and $M_{\pm}$ through direct calculation.

Following a series of lengthy elimination steps, we obtain the final effective medium result, which exhibits neither magnetic response nor magnetoelectric coupling—i.e., $\boldsymbol{\mu=I}$ and $\boldsymbol{\gamma=0}$. The resulting medium corresponds to a uniaxial crystal with a purely dielectric, diagonal response, and its permittivity tensor is given by:

(S4.11)

The corresponding dispersion relation is shown in Fig. 6(a) of the main text. The diagonal permittivity components can be tuned via $\omega_{c}$ and $\omega_{p}$, and remain independent of $\Lambda$. The transmission and reflection amplitude spectra calculated using these material parameters perfectly match that obtained from rigorous computations based on an extended transfer matrix method that includes all higher-order scattering modes [S4], as shown in Fig. 6 for different polarizations and Fig. S3 for different incident angles.


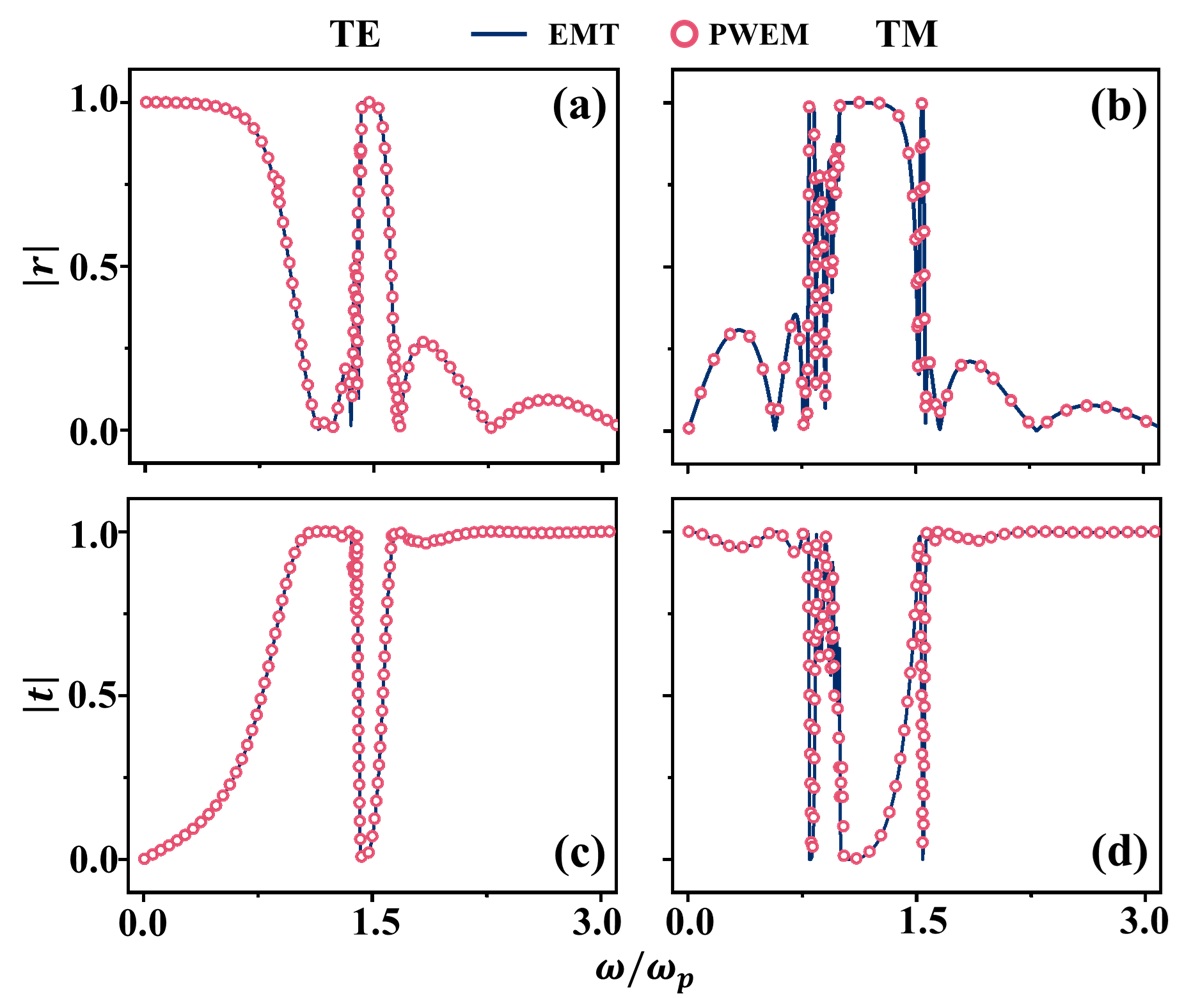


**Figure S3. Reflection and transmission amplitude spectra of the uniaxial medium under a rapidly twisted helical magnetic field at an incidence angle of $\boldsymbol{18^{\circ}}$.** (a-b) (or (c-d)) Reflection (or transmission) amplitude spectra of the homogeneous uniaxial medium for (a) (or (c)) TE and (b) (or (d)) TM polarizations, comparing results from the EMT model (lines) with global PWEM solutions (circles). The strength of the helical magnetic field is fixed at $\omega_{c}=\omega_{p}$.

Notably, the material response does not include any magnetoelectric coupling conventionally arising from the interaction between the electron gas and the uniform magnetic field. Consequently, the system is intrinsically non-chiral. The chirality originally associated with the WPs is completely removed in the $\Lambda\to\infty$ limit, as it is reconstructed and eliminated through the coupling between Fermi arc metal states of opposite chirality.

**References**

[S1] Wang D, Yang B, Gao W, et al. Photonic Weyl points due to broken time-reversal symmetry in magnetized semiconductor. Nat Phys 2019; 15: 1150–1155.

[S2] Gao WL, Yang B, Lawrence M, et al. Photonic Weyl degeneracies in magnetized plasma. Nat Commun 2016; 7: 12435.

[S3] Li ZF, Ma SJ, Li SW, et al. Observation of Copropagating Chiral Zero Modes in Magnetic Photonic Crystals. Phys Rev Lett 2025; 134: 033802.

[S4]  Hao JM, and Zhou L. Electromagnetic wave scatterings by anisotropic metamaterials: Generalized 4$\times$4 transfer-matrix method. Phys Rev B 2008; 77: 094201.
